# Phenotypic differences in the appearance of soft rice and its endosperm structural basis

**DOI:** 10.3389/fpls.2023.1074148

**Published:** 2023-02-01

**Authors:** Peng Fan, Jian Xu, Zhijie Wang, Guodong Liu, Zhenzhen Zhang, Jinyu Tian, Haiyan Wei, Hongcheng Zhang

**Affiliations:** Jiangsu Key Laboratory of Crop Cultivation and Physiology, Innovation Center of Rice Cultivation Technology in Yangtze Valley, Ministry of Agriculture, Co-Innovation Center for Modern Production Technology of Grain Crops, Yangzhou University, Yangzhou, China

**Keywords:** soft rice, appearance quality, endosperm structure, rice filling, formation mechanism

## Abstract

In view of the significant differences among genotypes in the appearance of soft rice, it is necessary to conduct research on the differences in the appearance quality of soft rice and their mechanisms. It can provide a theoretical basis for the selection and breeding of superior appearance varieties at a later stage. In order to clarify the differences in appearance phenotypes between different soft rice genotypes and structural basis of endosperm structures behind the differences, four soft rice varieties were selected in this study, including two varieties with good-appearance and two varieties with cloudy appearance. The differences in appearance phenotypes and endosperm structure in mature grains of soft rice with different appearance phenotypes were scientifically analyzed. The development process of their endosperm differences at the filling stage was investigated. The results show that the difference in the rice appearance of soft rice varieties mainly lay in the chalk-free seed transparency and chalkiness. These differences were caused by two completely different types of endosperm structure. Fewer and smaller starch grain cavities were responsible for higher chalk-free transparency of soft rice grains, denser starch granules arrangement caused lower chalkiness of soft rice grains. Ten days after flowering, the starch granules in the back and heart of good-appearance soft rice were already significantly fuller and more closely packed than those of cloudy soft rice. At the same time, the number and area of starch granule holes were significantly smaller than those of cloudy soft rice. This difference gradually increased until maturity. Therefore, based on appearance evaluation, soft rice with good-appearance should have higher transparency and lower chalkiness. The endosperm starch granules should be full and tightly arranged. The number of starch grain cavities and the area should be smaller. These differences develop in the early stages of grouting and gradually increase.

## 1 Introduction

Soft rice is a type of rice between glutinous and sticky rice in terms of amylose content (AC) (Wang et al., 2008; Fan et al., 2022), mainly distributed in Jiangsu Province, Shanghai, Anhui Province, Zhejiang Province and Yunnan Province. Soft rice generally has an AC between 8%-13% (Wang et al., 2022) and thus cooked soft rice usually has the characteristics of soft and sticky texture, high elasticity, less hardening after cooling, less retrogradation and excellent taste (Wang and Wang, 2002; Yu et al., 2012; Syahariza et al., 2014; Li et al., 2016). As of 2021, 56 soft rice varieties have been certified in the Yangtze River Delta region, including Jiangsu Province, Shanghai and Zhejiang Province (Gao et al., 2014). Due to their excellent palatability, soft rice varieties are expanding in the Yangtze River Delta region, with a planting area of more than 8.0 × 10^5^ hm^2^ in 2020 (Wang et al., 2021). Compared with traditional non-soft rice, soft rice has excellent palatability. However, the endosperm of soft rice commonly presents a translucent or cloudy appearance. This has become a major factor limiting its market competitiveness (Adegoke et al., 2021; Bian et al., 2021; Feng et al., 2021; Wang et al., 2022). In terms of rice appearance, although consumers in different regions have different preferences for rice grain shape due to cultural differences, they all generally prefer rice with low chalkiness and high transparency (Sreenivasulu et al., 2015; Wang et al., 2016).

Rice appearance quality is the specific appearance characteristics developed after the involvement of rice grain endosperm in light reflection and transmission. Starch, the most important storage material in rice endosperm, accounts for over 80% of the dry weight of endosperm. Endosperm structure (particularly the arrangement and morphological structure of starch grains in the endosperm) is an important factor affecting the appearance quality of rice (Wang et al., 2015; Nevame et al., 2018; Zhou et al., 2019; Zhu et al., 2020; Deng et al., 2021; Huang et al., 2021; Hao, 2021). It has been shown that there are inter-varietal differences in the appearance quality of non-soft rice, which are mainly reflected in the chalky phenotype. The chalky phenotype is developed mainly due to the loose arrangement of endosperm starch grains (Lin et al., 2016; Nevame et al., 2018; Zhou et al., 2019). Therefore, are there any differences in the appearance quality of different soft rice varieties? In what ways do these differences manifest themselves? What are the causes of these differences? These have rarely been studied. In this study, the differences in appearance quality were investigated based on soft rice varieties with significantly different in appearance quality. The causes of different appearance quality were analyzed based on endosperm structure. This may provide some references for improving soft rice quality.

## 2 Materials and methods

### 2.1 Test sites and rice varieties

The tests were conducted in 2021 at the test site in Shatou, Yangzhou, with wheat being the previous crop in that area. The good-appearance soft rice varieties (Yangnongxiang 28 and Nanjing 5718), and the cloudy soft rice varieties (Wuxiangjing 113 and Yangjing 5118), used in 2020, were selected as test materials. For notations, Y28 represents Yangnongxiang 28; N5718 represents Nanjing 5718; W113 represents Wuxiangjing 113; Y5118 represents Yangjing 5118.

### 2.2 Field management

The test site was selected from plots with medium to good ground strength and balance (basic ground strength), yields above 400 kg and easy access to irrigation. A normalized group design was used.

The cultivation method was machine inserting blanket seedlings, in rows 30cm × 11.7cm apart, and with 4-5 plants per hole.

Nitrogen fertilizer level was 270kg /hm^2^, based on base fertilizer: tiller fertilizer: spike fertilizer = 3.5:3.5:3. Tiller fertilizer was applied seven days after transplanting, and spike fertilizer was applied at the inverted four-leaf stage. The ratio of N: P: K was 2:1:2, with phosphate as a one-time base fertilizer and potash applied in equal amounts before plowing and at the nodulation stage respectively. Water management, pest and weed control and other related cultivation measures were implemented following high yield cultivation requirements.

### 2.3 Sample taking

Spikes with uniform growth and flowering on the same day were tagged at the beginning of flowering for each variety. Seed samples were taken 5, 10, 15 and 20 days after flowering (DAY) and at maturity.

### 2.4 Chalkiness

The chalkiness of the seeds at maturity was determined according to GB/T 17891-2017. The chalkiness, chalk area and chalky rate were calculated (WS-SC-E, China).

### 2.5 Transparency

When the rice sample was placed in a 1 cm thick cuvette, the transparency of the rice can be expressed by its transmission using a colorimeter with a D65 light source (CM-5 Japan). The transparency of normal seeds at maturity and non-chalky seeds. were measured.

### 2.6 Maturing seeds for somatic microscopy

Representative rice was arranged neatly in a dark room. The appearance of the white rice was photographed with a stereo microscope (Leica S8AP0, Germany).

### 2.7 Observations on the structure of the endosperm of rice seeds

The seed samples were fixed in pre-chilled 2.5% glutaraldehyde solution immediately after leaving the spike at the filling stage, and washed three times with 0.1 mol/L (pH 7.2) phosphate buffer after three hours. Then ethanol gradient dehydration, isoamyl acetate replacement, and CO2 critical point drying were conducted. The final sample was prepared using ion beam sputtering coating and then observed using a scanning electron microscope (Philips XL-30 ES-EM, Netherlands).

Mature seeds were frozen in liquid nitrogen for 3~5 min. Then the external force was applied to the rice grains with forceps to make them brittle. The sample was pasted, sputter-coated and observed in the same way as described above.

### 2.8 Starch particle size and distribution

The particle size distribution of starch was investigated using a laser diffraction particle size analyzer (Mastersizer 2000, Malvern, UK). About 0.2 g starch samples were immersed in 500 mL absolute ethyl alcohol and stirred at 2000 rpm. The instrument was calibrated to measure starch particle sizes ranging from 0.1 to 2000 µm.

### 2.9 Seed moisture distribution status

T2 (transverse relaxation time) values of cooked rice were measured using a nuclear magnetic resonance analyzer (NMI-20Analyst, Shanghai Niumag Electronic Technology Co., Shanghai, China) with the Carr-PurcellMeiboom-Gill (CPMG) sequence. A subsample of each rice sample used to determine taste was weighed to 5 g in glass vials and placed in a nuclear magnetic tube with a diameter of 25 mm. The center frequency was first calibrated with a free induction (FID) decay pulse train using the NMR spectrum analysis software. The parameters of the CPMG pulse train for each rice sample were set as follows: main frequency SF1=20MHz; sampling frequency SW=200 kHz; 90° hard pulse RF pulse width P1=6 μs; 180° hard pulse RF pulse width P2=13.04 μs; signal sampling points TD=600010; waiting time for repeat sampling, TW=2500 ms; the number of replicates, NS=4; echo number NECH=15000. This technique was studied and explained in depth by Klitsadee et al. (Klitsadee et al., 2019).

### 2.10 The number and area of starch granule holes

After obtaining the images by scanning electron microscopy, 200 starch grains were selected for each sample according to the magnification-corrected scale, and the number of starch grain cavities was calculated. The area of the cavities was measured using the PS software, and their mean and standard deviation were obtained using Excel.

### 2.11 Statistical analysis

Each experiment was repeated at least three times independently. All of the reported date were calculated into averages and analyzed using one-way analysis of variance (ANOVA) and Tukey’s test in SPSS (version 16.0).

## 3 Results and analysis

### 3.1 Differences in appearance quality of different soft rice varieties

**Figure 1:**
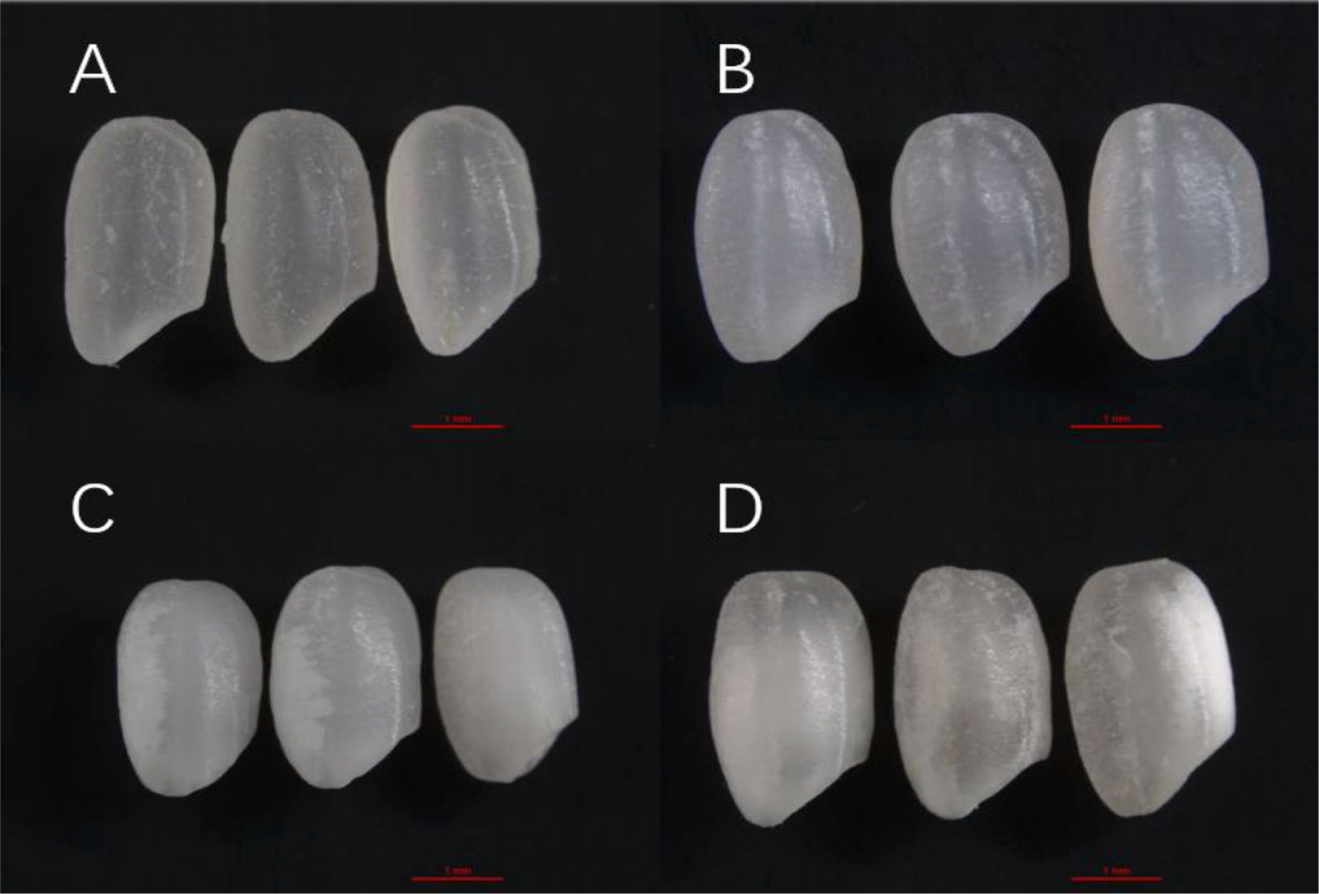
Images of rice grains from a stereo microscope: **(A)** Y28, **(B)** N5718, **(C)** W113 and **(D)** Y5118. The red line in **Figure 1** is a scale representing one millimeter in length.

The appearance of Y28 and N5718 differed significantly from that of W113 and Y5118 (**Figure 1**). Y28 and N5718 had transparent texture, and small chalky areas could be seen in the core and base of individual grains (**Figures 1A, B**). The endosperm of W113 and Y5118 generally showed a cloudy opaque phenotype.

Rice transparency is measured as the overall transparency of sampled fine rice grains in previous studies, which refers to light transmission performance of sampled fine rice under transmitted light. It can be affected by both chalky and non-chalky parts of rice grains. In order to further investigate the causes of the differences in soft rice transparency, the non-chalky grain transparency of four soft rice varieties was also measured in this study. This transparency can represent original chalk-free transparency of rice grains without the interference of chalkiness (**Table 1**).

**Table 1:** Differences in appearance quality indexes of different soft rice varieties.

Appearance	Variety	Moisture content (%)	Chalk-free seed transparency (%)	Transparency (%)	Chalky area (%)	Chalky rate (%)	Chalkiness (%)
Good-appearance	Y 28	14.21 a	12.00 a	11.10 a	26.41 d	17.15 d	4.53 d
	N5718	14.23 a	12.31 a	10.99 a	28.98 c	21.13 c	6.12 c
Cloudy	W113	14.19 a	10.15 b	7.68 b	33.74 a	74.23 b	22.04 b
	Y5118	14.25 a	10.87 b	7.79 b	31.61 b	80.53 a	25.46 a

For each column, values not displaying the same letter are significantly different (p < 0.05).

**Table 1** shows no differences in the moisture content of rice grains (**Table 1**). The good-appearance soft rice varieties had significantly higher chalk-free seed transparency than the cloudy soft rice varieties, indicating that the grains of the good-appearance soft rice varieties were originally more transparent. The transparency was 0.9%-1.32% and 2.97%-3.08% lower than their chalk-free seed transparency for the good-appearance soft rice varieties and the cloudy soft rice varieties, respectively. This indicates that rice grains of the cloudy soft rice varieties had lower transparency, higher chalkiness and larger differences in transparency from their chalk-free seed transparency. This should be influenced by the different chalky phenotypes, because chalky area, chalky rate and chalkiness of the good-appearance soft rice varieties were significantly lower than those of the cloudy soft rice varieties. This indicates that the good-appearance soft rice varieties had higher chalk-free seed transparency and lower chalkiness.

### 3.2 Differences in endosperm structure of mature grains of soft rice varieties with different appearance

The above analysis shows that the appearance of these four varieties was significantly different regarding chalk-free seed transparency and chalkiness. Normal and non-chalky grains of these four varieties were selected to further investigate the endosperm structure that caused differences in these two types of appearance.

#### 3.2.1 Cross-sectional electron micrographs of rice grains at maturity

**Figure 2:**
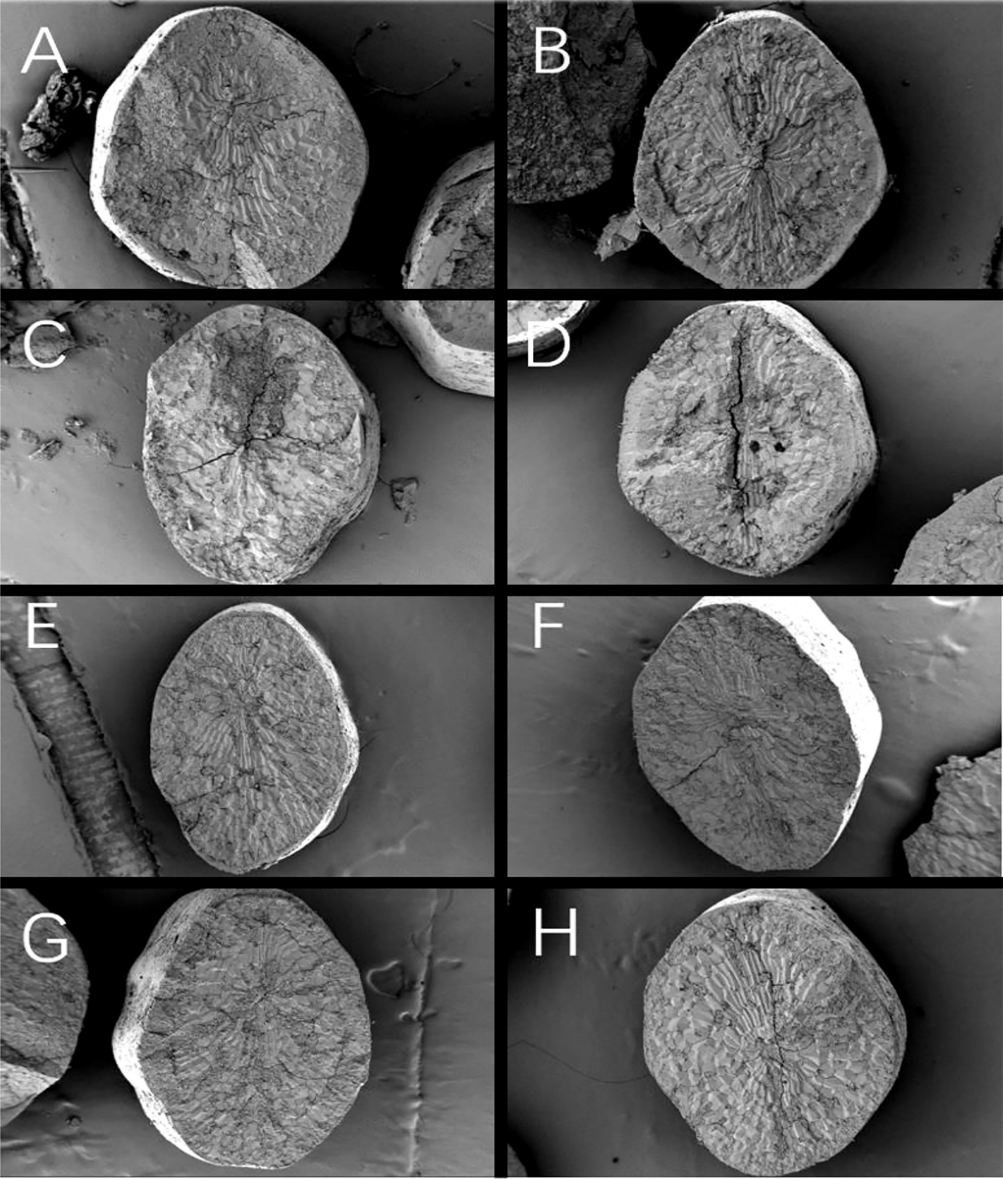
Cross-section of rice grains: normal grains of **(A)** Y28, **(B)** N5718, **(C)** W113and **(D)** Y5118; non-chalky grains of **(E)** Y28, **(F)** N5718, **(G)** W113 and **(H)** Y5118.

The endosperm of normal grains of Y28 and N5718 had relatively dense structures, and there was no clearly visibly structure at low magnification (**Figures 2A, B**). The endosperm of normal grains of W113 and Y5118 had a more visibly loose structure in the endosperm core and belly. Clear cracks occurred in the endosperm cross-section (**Figures 2C, D**). The endosperm of non-chalky grains of all four varieties had a denser structure, and there was no loose structure at low magnification (**Figures 2E–H**).

#### 3.2.2 Electron micrographs of endosperm of rice grains at maturity

**Figure 3:**
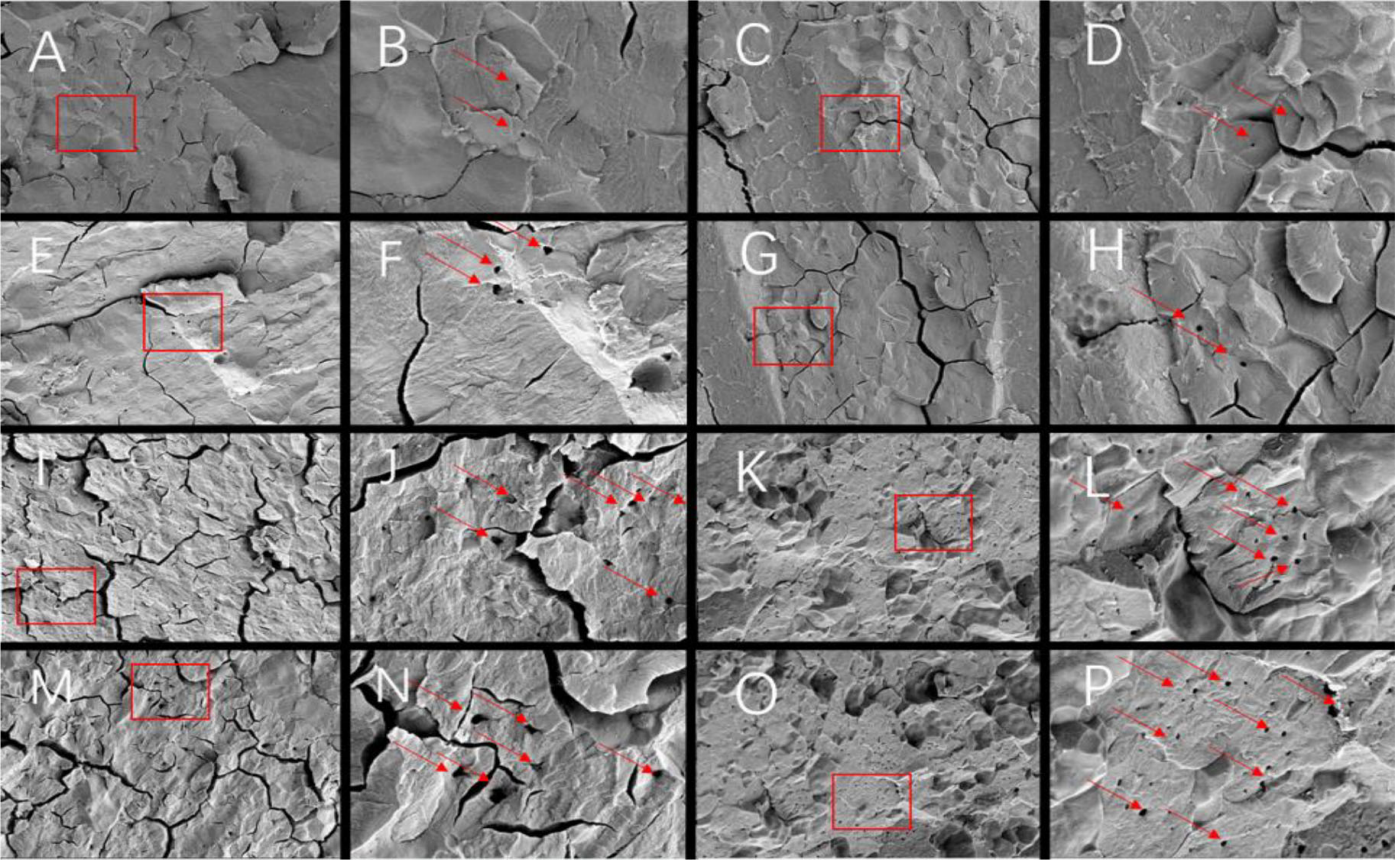
Endosperm back of **(A, B)** normal grains and **(C, D)** non-chalky grains of Y28, **(E, F)** normal grains and **(G, H)** non-chalky grains of N5718, **(I, J)** normal grains and **(K, L)** non-chalky grains of W113, **(M, N)** normal grains and **(O, P)** non-chalky grains of Y5118. Images **A/C/E/G/I/K/M/O** are at 1500x and Images **B/D/F/H/J/L/N/P** are at 5000x. Images **B/D/F/H/J/L/N/P** are the enlarged view of red rectangle areas in **A/C/E/G/I/K/M/O,** respectively.

**Figure 4:**
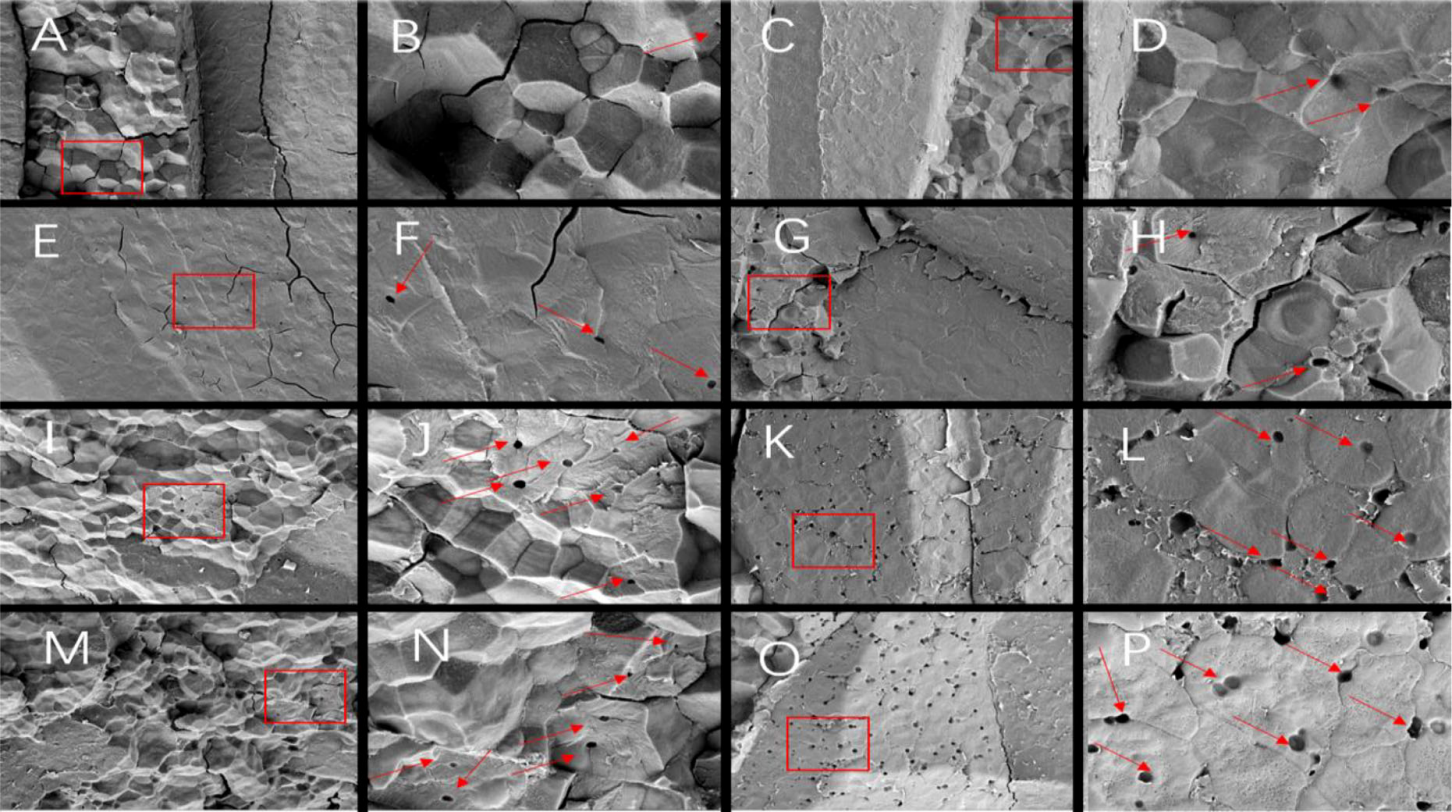
Endosperm core of **(A, B)** normal grains and **(C, D)** non-chalky grains of Y28, **(E, F)** normal grains and **(G, H)** non-chalky grains of N5718, **(I, J)** normal grains and **(K, L)** non-chalky grains of W113, **(M, N)** normal grains and **(O, P)** non-chalky grains of Y5118. **A/C/E/G/I/K/M/O** are at 1500x and **B/D/F/H/J/L/N/P** are at 5000x. **B/D/F/H/J/L/N/P** are the enlarged view of red rectangle areas in **A/C/E/G/I/K/M/O**, respectively.

**Figure 5:**
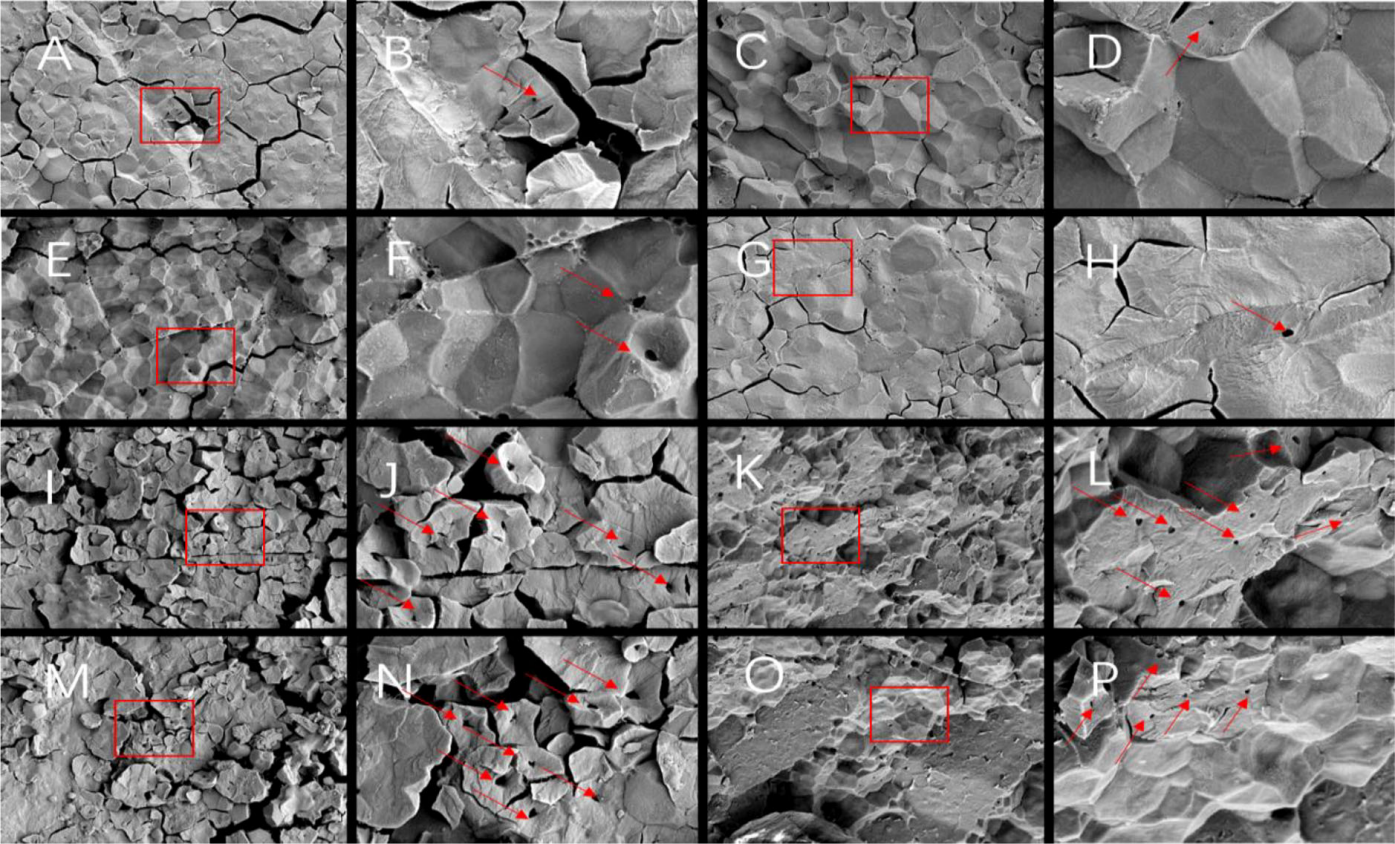
Endosperm belly of **(A, B)** normal grains and **(C, D)** non-chalky grains of Y28, **(E, F)** normal grains and **(G, H)** non-chalky grains of N5718, **(I, J)** normal grains and **(K, L)** non-chalky grains of W113, **(M, N)** normal grains and **(O, P)** non-chalky grains of Y5118. **A/C/E/G/I/K/M/O** are at 1500x and **B/D/F/H/J/L/N/P** are at 5000x. **B/D/F/H/J/L/N/P** are the enlarged view of red rectangle areas in **A/C/E/G/I/K/M/O**, respectively.

Since different endosperm structures often appear in the back, heart and belly of rice grains, this study compared the endosperm structure differences of soft rice varieties with different appearance types from these three parts. The endosperm back of normal grains of Y28 and N5718 had a dense structure, with fewer cracks and almost no single starch grains (**Figures 3A, E**). The endosperm back of normal grains of W113 and Y5118 were denser, without single starch grains (**Figures 3I, M**). However, there were more cracks which were mostly intergrain gaps of compound starch grains. The endosperm back of non-chalky grains of the four varieties was also dense (**Figures 3C, G, K, O**). At the heart of the endosperm, well-developed starch grains were densely arranged, polyhedral and angular in both normal and non-chalky grains of Y28 and N5718 (**Figures 4A, C, E, G**). The starch grains in the endosperm core of normal grains of W113 and Y5118 had different sizes and were unevenly arranged (**Figures 4L, M**). In contrast, the endosperm core of non-chalky grains was densely arranged (**Figures 4K, O**). The endosperm structure of the belly of normal grains of Y28 and N5718 was slightly loose, with clear intergrain gaps between the compound starch (**Figures 5A, E**). The belly endosperm of normal grains of W113 and Y5118 was looser. The starch grains had different sizes, and the gap between compound starch grains was larger. There were also some single starch grains (**Figures 5I, M**). In contrast, the belly endosperm structure of their non-chalky grains was dense (**Figures 5C, G**).

The magnified area of the endosperm back shows that all four soft rice varieties had cavities in the starch grains. It is also found that the number of cavities in the endosperm of the good-appearance soft rice varieties was significantly smaller than that of the cloudy soft rice varieties (**Figures 3B, D, F, H, J, N, P**). At the heart of the endosperm, a small number of cavities were observed on the amyloplast surface in the magnified area of the endosperm core of the good-appearance soft rice varieties (**Figures 4B, D, F, H**). In contrast, the endosperm core of the cloudy soft rice varieties had a large number of cavities. The cavities in the cloudy varieties can be divided into two types: the pore directly on the amyloplast surface and the pore at the bonding interface of amyloplasts and proteins (**Figures 4J, L, N, P**). The magnified area of the endosperm belly also shows that there were more cavities on dense starch grains of the cloudy varieties than that of the good-appearance soft rice varieties (**Figures 5B, D, F, H**). What is more consistent is that, in these three areas, the number of holes in the non-chalky seeds of the two good-appearance soft rice varieties was also low, while the number of holes in the non-chalky seeds of the two cloudy soft rice varieties was also high. Further comparison of the endosperm structure of mature rice grains of the four varieties shows that the number and size of cavities in the endosperm of normal and non-chalky grains of the same variety did not differ significantly, the number and size of cavities in the endosperm of good-appearance and cloudy varieties differ significantly (**Table 2**).

**Table 2:** Comparison of cross-sectional cavities of individual starch grains at maturity.

Appearance	Variety	Grain types	Number of cavities	Pore diameter /nm
Good-apprarance	Y28	Normal	0.19 b	593.29 c
		Non-chalky	0.20 b	589.42 c
	N5718	Normal	0.21 b	601.18 c
		Non-chalky	0.23 b	596.56 c
Cloudy	W113	Normal	1.18 a	873.06 b
		Non-chalky	1.23 a	869.56 b
	Y5118	Normal	1.25 a	912.16 a
		Non-chalky	1.19 a	910.42 a

For each column, values not displaying the same letter are significantly different (p < 0.05).

The endosperm differences between non-chalky and normal seeds were mainly reflected in the dense structure of all parts of the endosperm (**Figures 3**–**5**), indicating that the denseness of endosperm structure (rather than the cavities) was the direct cause of the chalky phenotype of soft rice. The differences in the appearance of chalk-free seeds of different varieties were mainly in transparency, and their corresponding differences in endosperm structure were mainly in the size and number of starch grain cavities (**Tables 1**, **2**). This indicates that the number and size of holes are directly responsible for the transparency of soft rice.

Compared with the cloudy varieties, the good-appearance soft rice varieties had denser endosperm structure (particularly in the endosperm belly). This suggests that the denseness of endosperm structure is the direct cause of the differences in chalky phenotypes of different soft rice varieties. The number and size of cavities in the endosperm of the good-appearance soft rice varieties were significantly smaller than those of the cloudy soft rice varieties (**Table 2**). This indicates that the cavities are the direct cause of the differences in the chalk-free seed transparency phenotypes of the different soft rice varieties.

#### 3.2.3 Starch granules size and distribution of rice grains at maturity

**Figure 6:**
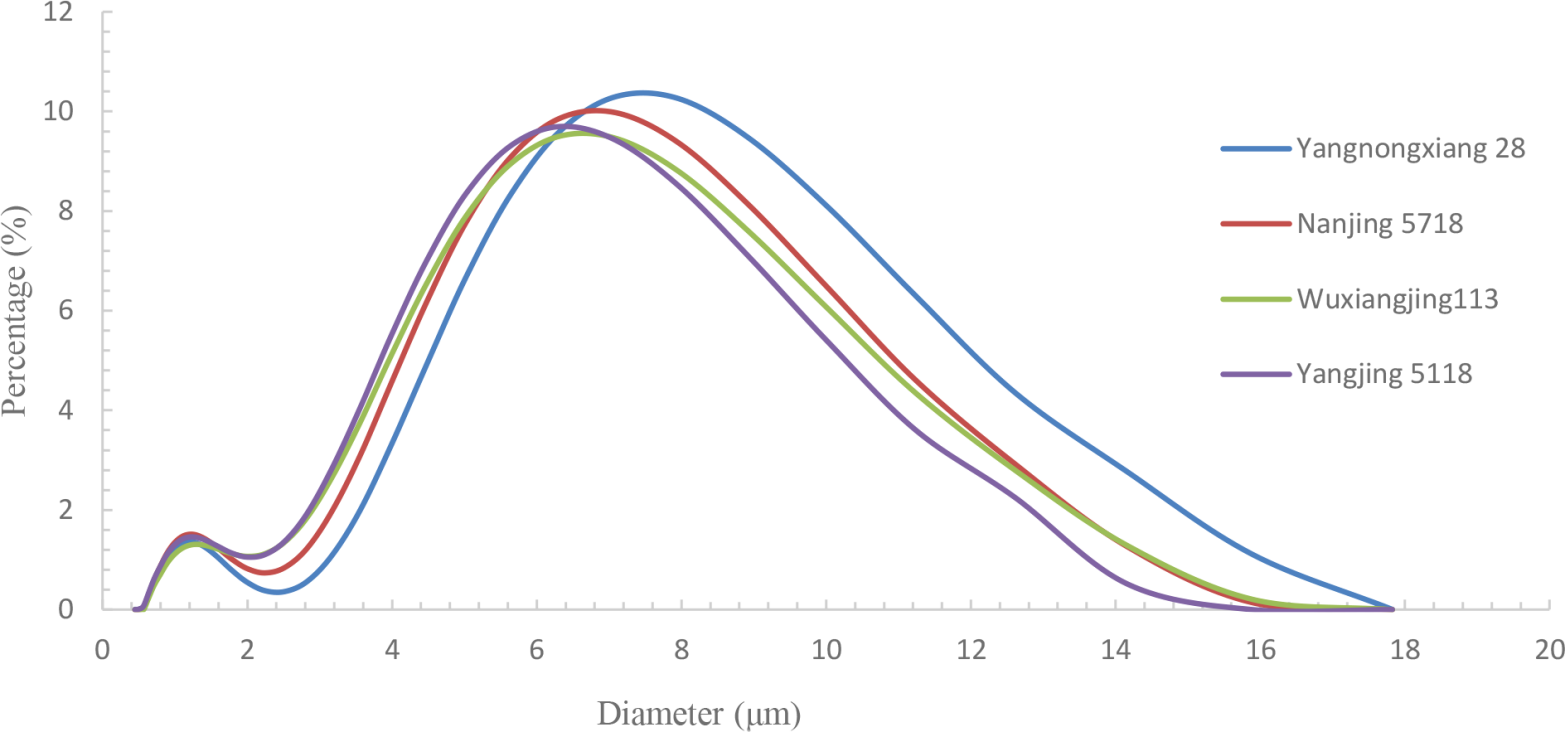
Starch grain size distribution of the four soft rice varieties.

The overall starch grain surface area and volume of Y28 and N5718 were larger than those of W113 and Y5118 (**Table 3**). In the starch grain size range of 0-2 μm, there was no apparent trend among the varieties. In the starch grain size range of 2-6 μm, the proportion of Y28 and N5718 was lower than that of W113 and Y5118, and significant levels were reached at 2-4 μm. While in the starch grain size range of 6-15 μm, the proportion of Y28 and N5718 was significantly higher than that of W113 and Y5118 (**Figure 6**). This indicates that the average starch grain fullness of the good-appearance soft rice varieties was higher than that of the cloudy soft rice varieties.

**Table 3:** Starch grain size distribution of the four soft rice varieties.

Appearance	Varieties	Surface area of starch grains (μm^2^)	Volume of starch grains (μm^3^)	0-2 μm	2-4 μm	4-6 μm	6-8 μm	8-15 μm
Good-apprarance	Y28	4.18 a	6.66 a	10.22 c	7.74 d	24.89 b	25.47 a	31.68 a
	N5718	3.82 b	5.94 b	11.81 b	12.54 c	28.21 a	24.27 b	23.17 b
Cloudy	W113	3.76 bc	5.83 c	10.75 c	15.95 b	28.38 a	22.96 c	21.96 c
	Y5118	3.57 c	5.54 d	12.06 a	16.92 a	29.80 a	22.69 c	18.53 d

For each column, values not displaying the same letter are significantly different (p < 0.05).

#### 3.2.4 Moisture distribution of rice grains at maturity

The transverse relaxation time is divided into short transverse relaxation time (T21, 0.1-10 ms) and long transverse relaxation time (T22, 10-100 ms). T21 and T22 represent the bound water and free water distribution inside the specimen, respectively. A2 represents a significant linear relationship between the signal amplitude of the magnetic resonance transverse relaxation spectrum and the moisture content of the grains. C2 represents the proportion of water in each state to the total moisture content of the grains. Compared with the cloudy varieties, the good-appearance varieties had significantly higher A22 and C22 but significantly lower C21 (**Table 4**). This indicates that the content and proportion of free water in the endosperm of the good-appearance soft rice varieties were significantly higher than those of the cloudy varieties.

**Table 4:** Moisture distribution state indicators of the four varieties.

Appearance	Varieties	T21 (ms)	T22 (ms)	A21(A.U.)	A22(A.U.)	C21(%)	C22(%)
Good-appearance	Y28	0.67 a	78.38 a	2208.91 a	50.65 a	97.76 c	2.24 b
	N5718	0.51 d	71.69 d	1784.69 b	45.49 b	97.51 d	2.49 a
Cloudy	W113	0.58 c	73.08 c	2075.63 a	38.77 c	98.17 b	1.83 c
	Y5118	0.62 b	76.57 b	2187.33 a	29.62 d	98.67 a	1.33 d

For each column, values not displaying the same letter are significantly different (p < 0.05).

### 3.3 Differences in endosperm structure at the filling stage

The differential endosperm structure of different soft rice varieties is closely related to the grain-filling process. What characteristics does endosperm exhibit during this process? Therefore, the endosperm structure of different soft rice varieties at different stages after flowering was systematically investigated.

#### 3.3.1 Endosperm structure at five days after flowering

**Figure 7:**
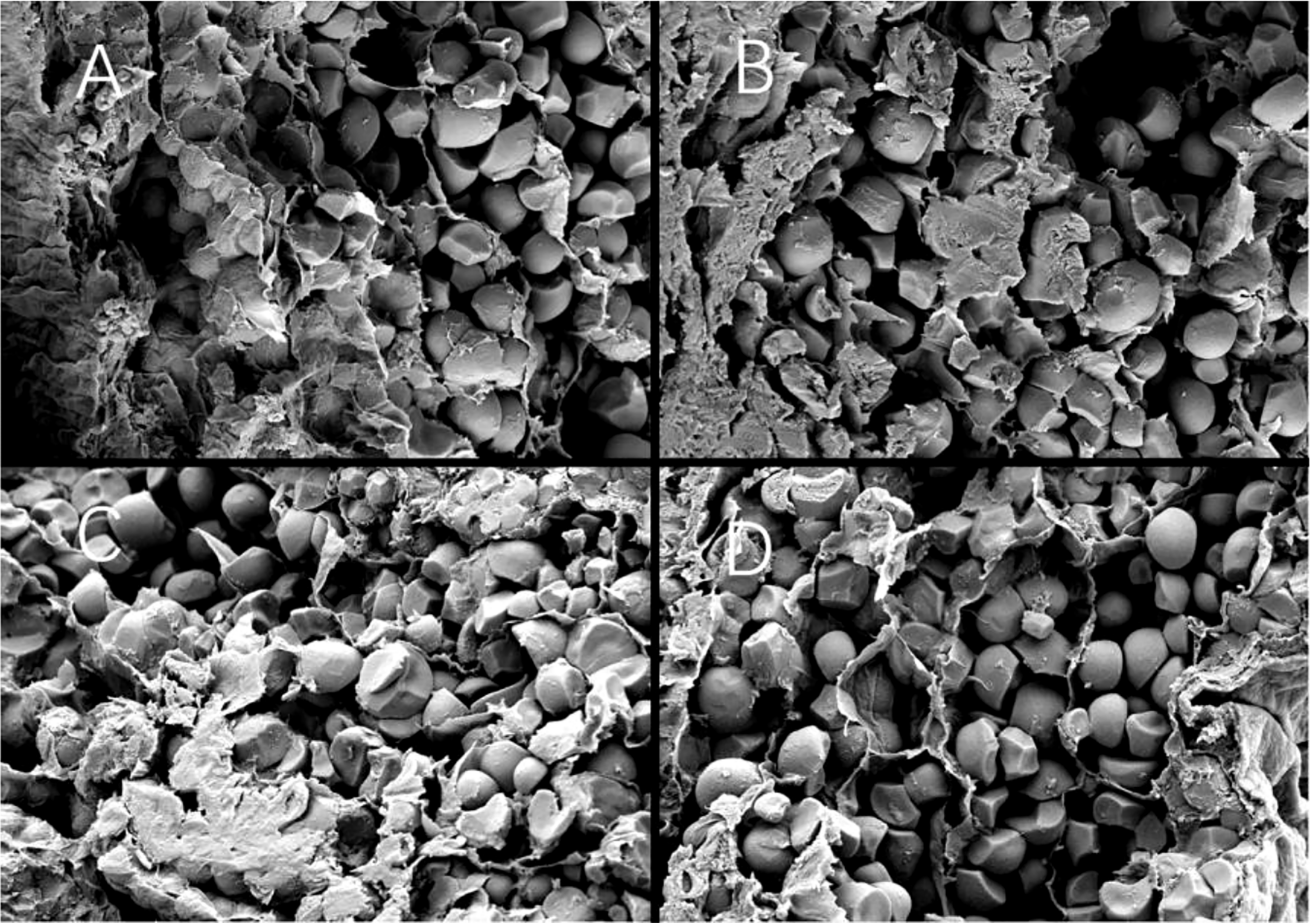
**(A)** Y28, **(B)** N5718, **(C)** W113 and **(D)** Y5118 (at 2500x).

At DAF 5, the grains started to accumulate starch and amyloplasts of the endosperm cells began to produce small starch grains. The starch grains were ovoid or spherical and less angular (**Figure 7**). At this point there were no significant differences between the varieties.

#### 3.3.2 Endosperm structure ten days after flowering

**Figure 8:**
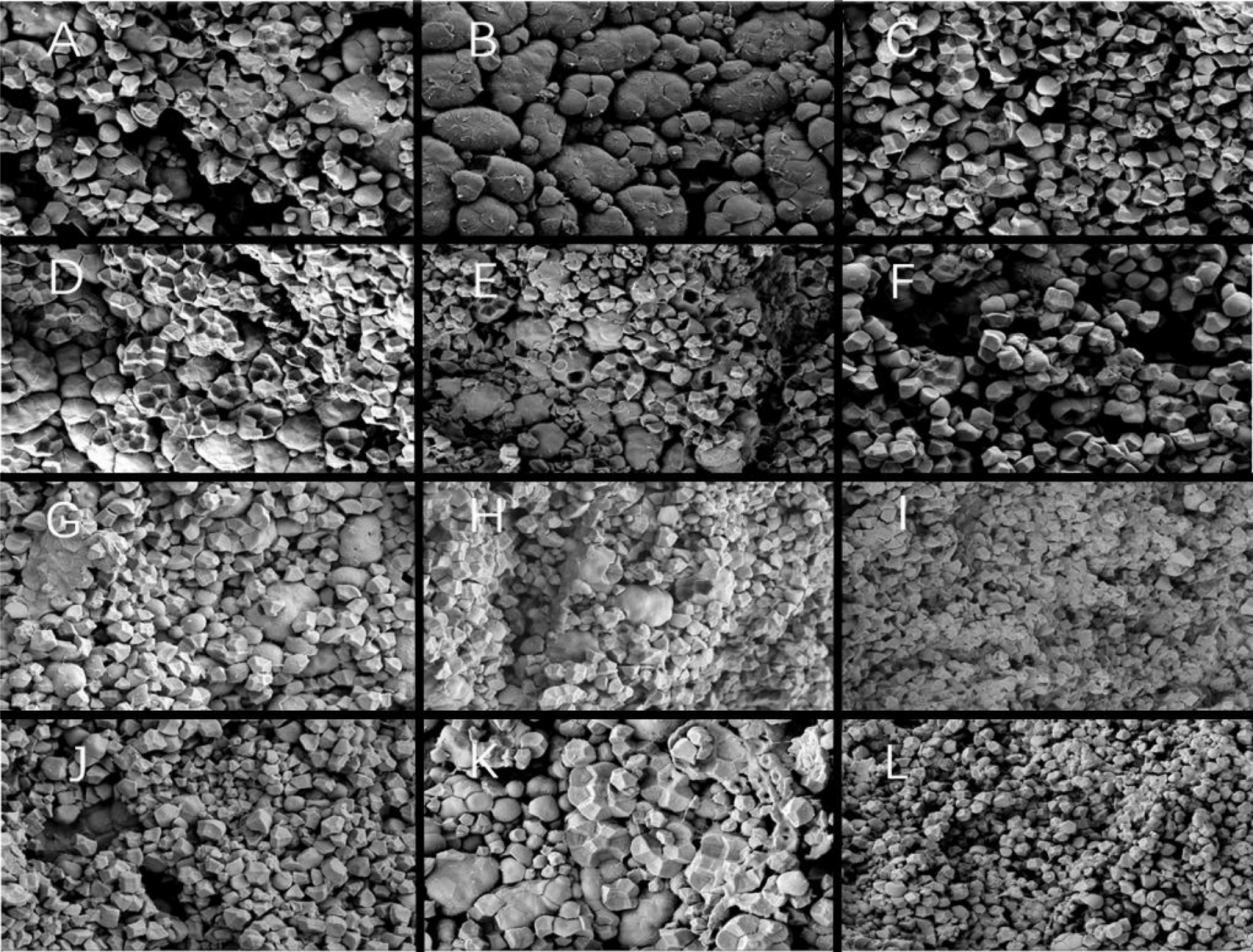
**(A)** Back, **(B)** core and **(C)** belly of Y28; **(D)** back, **(E)** core and **(F)** belly of N5718; **(G)** back, **(H)** core and **(I)** belly of W113; **(J)** back, **(K)** core and **(L)** belly of Y5118 (at 1500x).

A large number of compound starch grains and a small number of single starch grains were observed in the endosperm back and core parts of Y28 and N5718. The compound starch grains were closely arranged and compressed. Spherical protein bodies can be observed between the starch grains (**Figures 8A, B, D, E**). The endosperm back and core parts of W113 and Y5118 had a small number of compound starch grains and a large number of single starch grains. The proportion of compound starch grains of W113 and Y5118 was significantly smaller than that of Y28 and N5718 (**Figures 8G, H, J, K**). The abdominal structure of the four varieties was looser than the back and core structures. The compound starch grains were almost invisible in the belly part, with many loose polyhedral single grains. These did not differ significantly among varieties (**Figures 8C, F, I, L**). At DAF 10, the endosperm structure of the different soft rice varieties showed differences between the back and core parts.

#### 3.3.3 Endosperm structure 15 days after flowering

**Figure 9:**
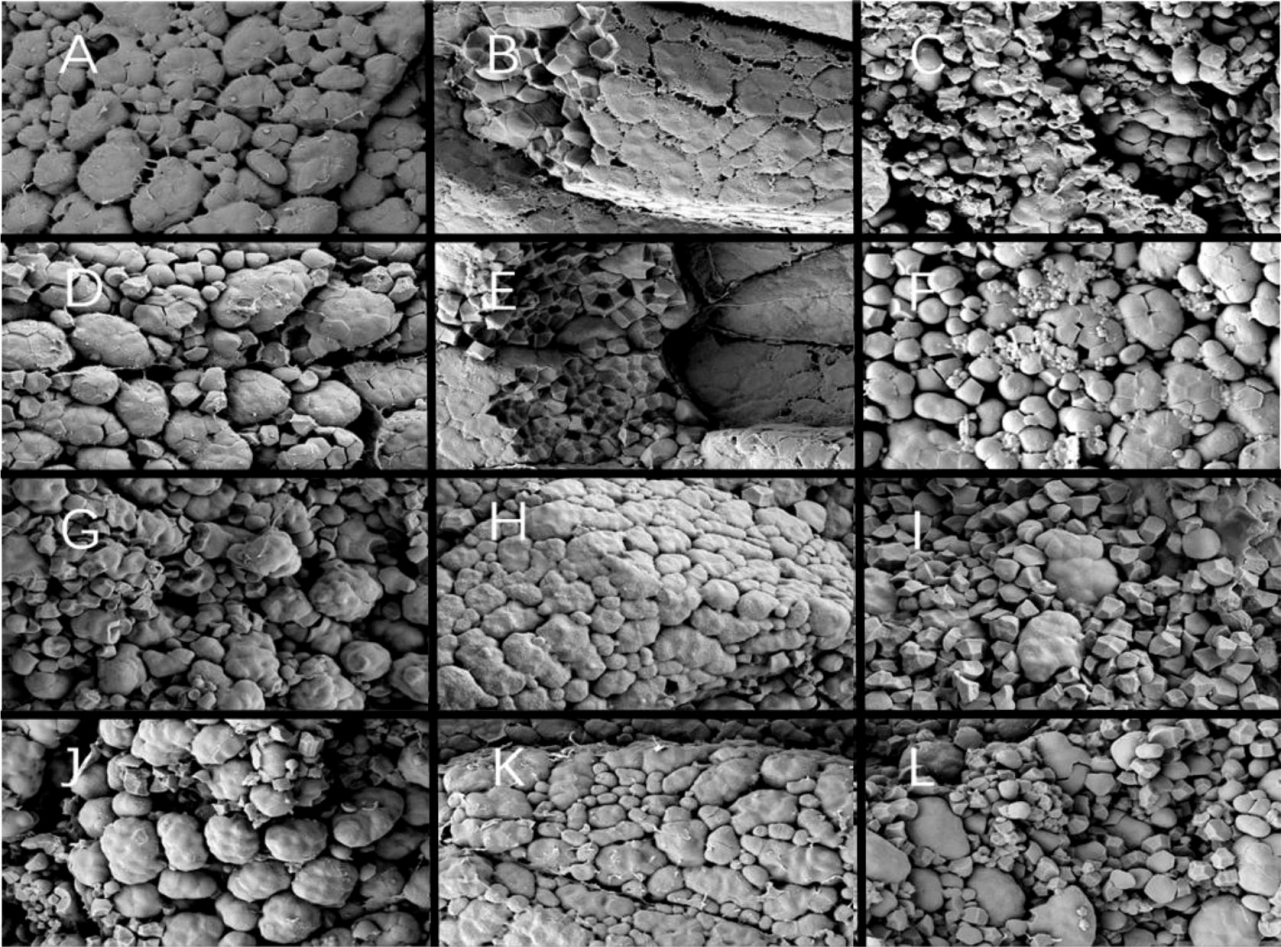
**(A)** Back, **(B)** core and **(C)** belly of Y28; **(D)** back, **(E)** core and **(F)** belly of N5718; **(G)** back, **(H)** core and **(I)** belly of W113; **(J)** back, **(K)** core and **(L)** belly of Y5118 (at 1500x).

Since DAF 15, the endosperm starch grains of each variety expanded and the gap was significantly reduced. The endosperm cells had been filled with amyloplasts, with deformed protein bodies remaining in the amyloplast gap (**Figure 9**). There were no clear differences between starch grains at the endosperm back of each variety. Single starch grains were no longer visible (**Figures 9B, E, H, K**). At the endosperm core, the starch grains of W113 and Y5118 showed more shrinkage than those of Y28 and N5718, without significant overall differences (**Figures 9A, D, G, J**). The endosperm back of each variety was looser. The endosperm belly of Y28 and N5718 had more compound starch grains, while that of W113 and Y5118 mainly had single starch grains (**Figures 9C, F, I, L**). At DAF 15, the endosperm structure of different soft rice varieties mainly varied in the belly part.

#### 3.3.4 Endosperm structure 20 days after flowering

**Figure 10:**
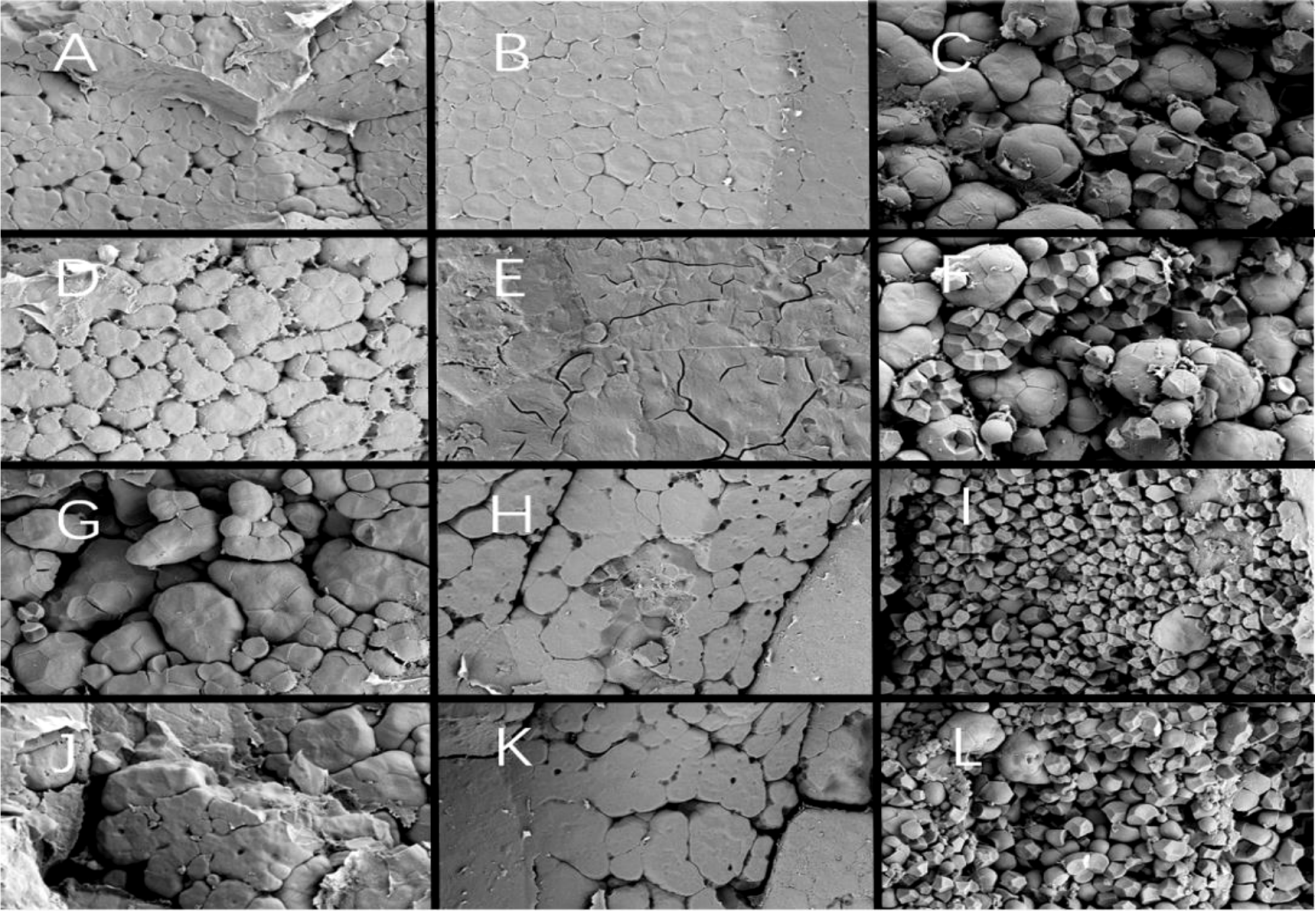
**(A)** Back, **(B)** core and **(C)** belly of Y28; **(D)** back, **(E)** core and **(F)** belly of N5718; **(G)** back, **(H)** core and **(I)** belly of W113; **(J)** back, **(K)** core and **(L)** belly of Y5118 (at 1500x).

At DAF 20, the core and belly parts of the endosperm of all soft rice varieties showed clear cavities. The back and core parts of the endosperm of all soft rice varieties were further filled compared to at 15 days. However, the gap between starch grains on the back was slightly larger than that on the core. This is possibly due to more protein bodies between the starch grains of the endosperm back, thus inhibiting the denser arrangement of the starch grains (**Figures 10A, B, D, E, G, H, J, K**). The gap between the endosperm back and core of the starch grains of W113 and Y5118 was larger than Y28 and N5718. Rounded compound starch grains occurred on the back of W113 and Y5118, but the round starch grains are no longer visible on the back of Y28 and N5718. It indicates that the fullness of W113 and Y5118 was lower than that of Y28 and N5718 at back and core parts (**Figures 10A, B, D, E, G, H, J, K**). The endosperm belly of Y28 and N5718 mainly had compound starchy grains, with a large intergrain gap. The endosperm belly of W113 and Y5118 had loose structure and a certain proportion of single starch grains (**Figures 10C, F, I, L**). Overall, there were clear differences in all parts of the endosperm of two appearance types of soft rice at 20 days after flowering.

#### 3.3.5 Development and differences in the pore structure of starch grains in soft rice

**Figure 11:**
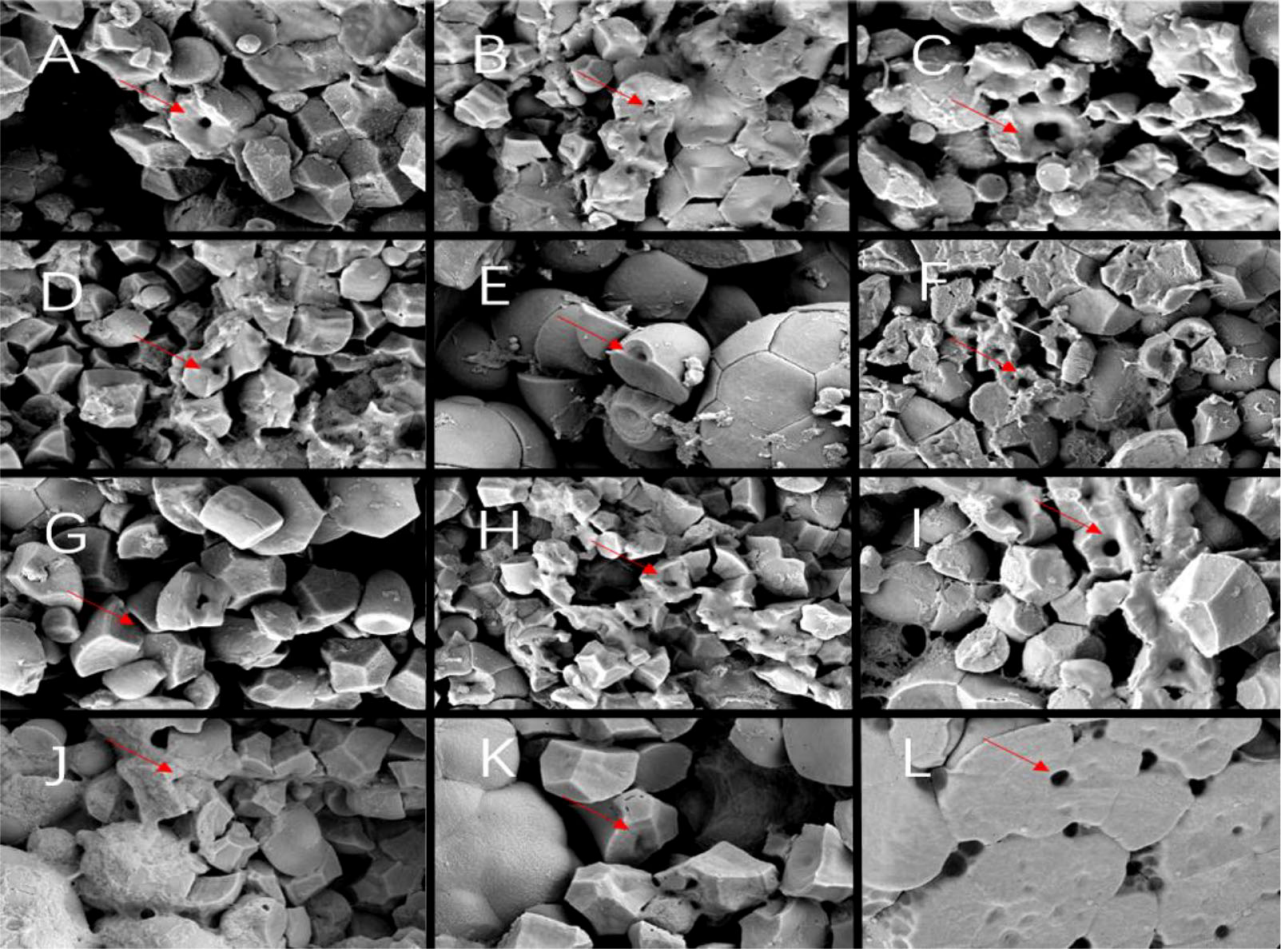
**(A)** 10d, **(B)** 15d and **(C)** 20d of Y28; **(D)** 10d, **(E)** 15d and **(F)** 20d of N5718; **(G)** 10d, **(H)** 15d and **(I)** 20d of W113; **(J)** 10d, **(K)** 15d and **(L)** 20d of Y5118 (at 5000x).

**Figure 12:**
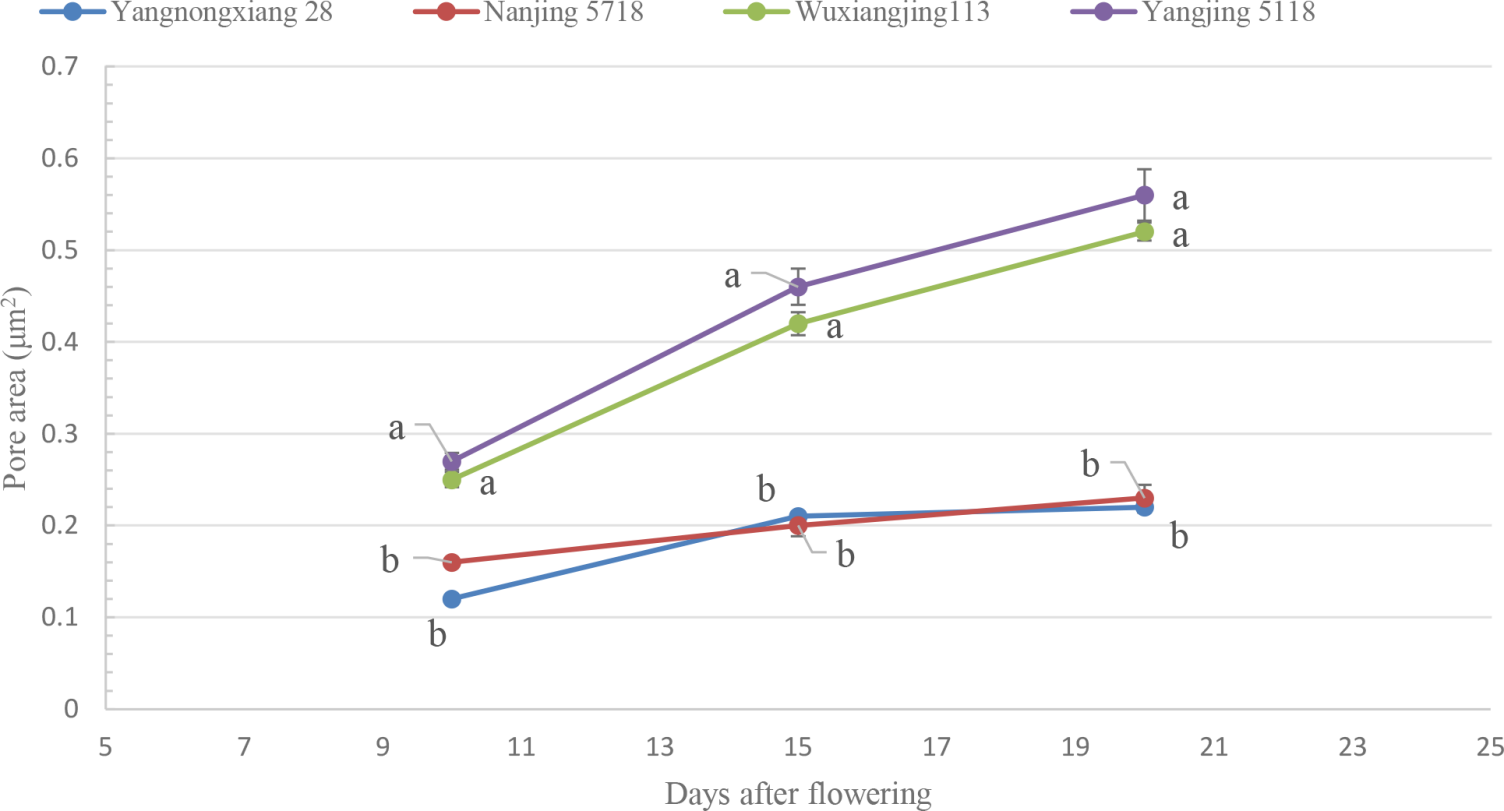
Dynamic variations of pore areas of individual starch grains of different soft rice varieties during the filling period. For each column, values not displaying the same letter are significantly different (p < 0.05).

At DAF 10, the starch grains in the endosperm of all soft rice varieties showed clear cavities. At each stage after DAF 10, cavities in the starch grains were also observed (**Figure 11**). This indicates that cavities were developed at DAF 10 at least or earlier. Before DAF 20, most of the cavities were still difficult to observe due to many of cell remnants in the endosperm. Thus, it was not feasible to accurately compare the number of cavities of the different soft rice varieties. Comparing the area of starch grain cavities at different stages shows that the area of starch grain cavities increased during DAF 10-20 for all varieties (**Figure 12**). This indicates that the diameter of starch grain cavities showed a dynamic increase during the filling period. It was also found that the area of starch grain cavities was significantly smaller in good-appearance soft rice than in cloudy soft rice in all periods.

## 4 Discussion

### 4.1 Appearance characteristics of good-appearance soft rice

Better appearance quality has always been an effective means for rice resources to enhance their market competitiveness. However, there is no industry-accepted standard for determining good-appearance soft rice varieties. In China, the appearance quality of ordinary rice is mainly measured by chalkiness (Li and Zhen, 2022). It indicates that lower chalkiness represents better rice appearance quality in the existing industry standards. In this experiment, it was found that similar to non-soft rice japonica rice, different appearance types of soft rice differed significantly in chalkiness. It shows that chalky differences are common to both soft and non-soft rice, and that less chalky soft rice also means having better appearance quality. However, differ from non-soft rice, soft rice tends to have a low transparency phenotype, which seriously affects the appearance quality of soft rice (Lin et al., 2016; Li et al., 2018; Zhang et al., 2019a). Li et al. found that soft rice varieties Nanjing 46 and Guangdong 194 showed a significant decrease in grain endosperm transparency at a storage moisture content below 12% (Li et al., 2018). Zhang et al. studied the transparency of dried brown rice with the same genotype but different AC and found that the transparency of rice with the AC of soft rice was significantly lower than that of normal rice (Zhang et al., 2019a). Wu et al. found that the endosperm of all low AC materials (including early indica rice varieties (lines), restorer lines and sterile male lines of hybrid rice) showed easily identifiable cloudy traits under indoor storage conditions for three months. However, the appearance of the endosperm of the varieties with medium or high AC did not change significantly and remained transparent (Wu et al., 2001). The transparency of different appearance types of soft rice was also found to be significantly different in this study, indicating that transparency is not only a trait that distinguishes soft rice from non-soft rice, but also varies greatly between different soft rice. The chalk-free seed transparency and chalkiness differed significantly between different soft rice varieties. Compared with the cloudy soft rice varieties, the good-appearance soft rice varieties had lower chalkiness but higher transparency. Therefore, the appearance quality of soft rice should be measured with chalkiness and transparency-related indexes.

### 4.2 Endosperm structural characteristics of good-appearance soft rice

The endosperm structure is a direct cause of the rice appearance quality. In many previous studies, the endosperm structure of highly chalky rice was compared with that of low chalky rice. It was found that chalkiness was mainly caused by the insufficient filling of endosperm, insufficient fullness of starch grains and loose arrangement (Dong et al., 2006; Zhang et al., 2006; Liu et al., 2007; Deng et al., 2021; Wang et al., 2021). In some transgenic rice studies, by comparing the endosperm structure of the chalky mutant with that of the wild type, it was found that the starch arrangement of the chalky mutant endosperm was more lax (Wang et al., 2010; Zhu et al., 2018; Can et al., 2020; Wu et al., 2022). In this study, we found that the chalky phenotype of soft rice was also due to the loose arrangement of endosperm starch grains, and the low chalky soft rice had fuller starch grains and a tighter starch grain arrangement structure. This was consistent with previous studies. In the starch granules size and distribution of rice grains test, it was also found that the average starch grain size was larger in the low chalky soft rice, and this result also confirmed the phenomenon that fuller starch grains contribute to the reduction of chalkiness. This may be due to the fact that fuller starch grains can easily compress to form a tight endosperm structure, and gaps between starch grains are less likely to exist.

The endosperm structure of soft rice was also compared with that of ordinary rice in previous studies (Lin et al., 2018; Shun et al., 2019; Yu et al., 2019). Zhang et al. analyzed the relationship between transparency and endosperm structure of starch grains of transgenic rice that was constructed based on glutinous rice and had different AC (Zhang et al., 2019a). They found many cavities between endosperm starch grains of glutinous rice. The pore number decreased with increasing AC, while rice transparency was enhanced. This indicates that the opacity of glutinous rice and the translucency of soft japonica rice were attributed to the cavities between starch grains. These cavities caused light scattering, thus affecting rice transparency (Zhang et al., 2013; Zhang et al., 2017). Hao et al. found that the number of cavities in individual starch grains was significantly higher in the low AC rice group compared to the medium and high AC rice groups (Hao, 2021). In the study of the Wx^IV^ gene, the presence of starch granule cavities in the endosperm of low transparency was found (Zhang et al., 2019b). However, these studies ignored the effect of chalk on transparency or only compared the endosperm transparency of individual seeds. In this study, the effect of chalkiness on transparency was well excluded by using chalk-free seed transparency as the focus indicator. It was found that starch grain cavities were responsible for the transparency of soft rice without chalky seeds. Combined with the previous studies, it is suggested that the fewer and smaller starch grain cavities contributed to the higher transparency of soft rice. Previous studies have found that the effect of moisture content on transparency were significantly and positively correlated with the size of starch grain cavities (Zhang et al., 2019a; Wang et al., 2021; Zhu et al., 2021; Hao, 2021). There are fewer studies on the moisture distribution state of rice grains on rice appearance. The relaxation time (T2) is closely related to the type and state of hydrogen protons, and the value of the transverse relaxation time reflects the water freedom inside the specimen (Song et al., 2016; Klitsadee et al., 2019; Yifu et al., 2019). This study found that the content and proportion of free water in good-appearance soft rice varieties were significantly higher than that in the cloudy soft rice varieties at the same moisture content between 14%-15%. The authors suggest that during rice grain drying to reduce moisture content, the water in the starch grain cavities was more easily precipitated than the semi-free water tightly bound to the starch. The cloudy soft rice lost more semi-free water due to more and larger starch grain cavities. The cavities that lost water scattered light and thus reduced endosperm transparency. This may be the pathway by which starch grain cavities affect the transparency of soft rice.

### 4.3 Development of endosperm structural differences

Many scholars have concluded that the development of rice chalkiness was closely related to the dynamic variation of dry matter accumulation, filling rate and moisture content of rice grains during rice filling (Yang et al, 2003; Liu et al., 2007; Wenting et al., 2021). The presented study found that at DAF 10, there were significant differences in the endosperm back and core. This indicates that differences in the endosperm structure of different soft rice varieties started to develop in the early filling stage. The differences were mainly in the back and core, possibly because from DAF 5 to 10, the filling material of all soft rice varieties in the early stage of filling was delivered more to the back and core parts and less to the belly part. This also shows that the good-appearance soft rice varieties had a higher filling rate than the cloudy soft rice varieties. At DAF 15, the difference in endosperm structure was mainly in the endosperm belly. This indicates that during DAF 10-15, more filling material was delivered to the belly and the belly structure rapidly increased in densitye after the endosperm cells at the back and core were filled. Thus, the difference in the endosperm structure between good-appearance soft rice varieties and cloudy soft rice varieties was further increased. At DAF 20, the differences between the endosperm of different rice varieties were evident in all endosperm parts. Possibly, in the middle and late filling stages, the filling efficiency of the good-appearance soft rice varieties was still higher than cloudy soft rice, and all parts of the endosperm were further filled. This suggests that the endosperm structure responsible for the chalky differences in soft rice starts to form at least ten days after filling and that the differences increase dynamically.

Yang et al. reported that the formation of starch grain cavities may be due to gradual water loss in the transition to the yellow ripeness stage after the number and shape of starch grains are set and filled with endosperm cells in the milky ripeness stage (Yang, 2015). Hao et al. conducted a structural analysis of the dynamic development cross-section of starch grains. They found that the cavity area of the single starch grain in the endosperm was formed at about DAF 4. It generally increased in size from DAF 4 to 20. At DAF 25-30, the cavity area of the single starch grain was stabilized (Hao, 2021). In this study, the cavities were not observed at DAF 5. However, at DAF 10, cavities occurred in the starch grains which was observed later than that of Hao et al (Hao, 2021). This may be due to the difference in the test varieties or the cell wall hindering ang observation. The results at least indicate that starch grain cavities occur before yellow ripeness stage. During DAF 10-20, these cavities showed a dynamic increase, which is consistent with the findings of Hao et al (Hao, 2021). This suggests that the endosperm structure, which causes differences in soft rice transparency, also begins to form at least ten days after filling. In conclusion, in addition to the selection of soft rice varieties with good appearance, the structural differences affecting the appearance quality of soft rice have already begun to form in the early filling stage. Therefore, reasonable fertilizer and water management should be ensured from the filling early stage in order to achieve a good filling rate. Thus, the endosperm can develop closely and ultimately promote the formation of good-appearance quality of soft rice.

## 5 Conclusions

This study concluded that the difference in the appearance of different soft rice varieties was mainly in chalk-free seed transparency and chalkiness. The soft rice varieties with good-appearance had higher chalk-free seed transparency and lower chalkiness. The analysis of endosperm structure indicates that the appearance difference was attributed to two completely different types of endosperm structure: fewer and smaller starch grain cavities were responsible for higher chalk-free seed transparency of soft rice, the denser starch grain arrangement caused lower chalkiness of soft rice. The analysis of endosperm development shows that the differences in endosperm structure of different soft rice varieties started to develop at DAF 10 until maturity. Then, the endosperm structure showed a continuous trend of development and enlargement process.

## Data availability statement

All relevant data is contained within the article: The original contributions presented in the study are included in the article/supplementary material, further inquiries can be directed to the corresponding authors.

## Author contributions

Writing—original draft preparation: PF. Consulting references: JX, ZW, JT, GL, ZZ. Funding acquisition: HW and HZ. All authors contributed to the article and approved the submitted version.
